# The conserved role of miR-2 and novel miR-109 in the increase in fecundity of *Diaphorina citri* induced by symbiotic bacteria and pathogenic fungi

**DOI:** 10.1128/mbio.01541-24

**Published:** 2024-10-07

**Authors:** Xiaoge Nian, Shujie Wu, Jielan He, Paul Holford, George Andrew Charles Beattie, Desen Wang, Yijing Cen, Yurong He, Songdou Zhang

**Affiliations:** 1 National Key Laboratory of Green Pesticide, Department of Entomology, College of Plant Protection, South China Agricultural University, Guangzhou, China; 2 School of Biology and Agriculture, Shaoguan University, Shaoguan, China; 3 School of Science, Western Sydney University, Penrith, Australia; 4 Department of Entomology and MOA Key Lab of Pest Monitoring and Green Management, College of Plant Protection, China Agricultural University, Beijing, China; Max Planck Institute for Chemical Ecology, Jena, Germany

**Keywords:** *Candidatus *Liberibacter asiaticus, *Cordyceps fumosorosea*, krüppel-homolog 1, juvenile hormone, microRNAs

## Abstract

Infection with pathogens can increase the fecundity and other fitness-related traits of insect vectors for their own advantage. Our previous research has reported the pivotal role of *DcKr-h1* in the fecundity improvement of *Diaphorina citri* induced by the bacterium, “*Candidatus* Liberibacter asiaticus” (*C*Las), and the fungus, *Cordyceps fumosorosea* (*Cf*). However, the posttranscriptional regulation of this process remains poorly understood. Given the significance of miRNAs in gene regulation, we delved into their roles in shaping phenotypes and their underlying molecular mechanisms. Our results indicated that two miRNAs, miR-2 and novel-miR-109, jointly inhibited *DcKr-h1* expression by binding to its 3′ untranslated region (UTR). In the *D. citri-C*Las interaction, the expression levels of miR-2 and novel-miR-109 in the ovaries of *C*Las-positive psyllids were lower compared to *C*Las-negative individuals. Overexpression of miR-2 or novel-miR-109 significantly decreased fecundity and *C*Las titer in ovaries and caused reproductive defects reminiscent of *DcKr-h1* knockdown. Similarly, in the *D. citri-Cf* interaction, the levels of miR-2 and novel-miR-109 markedly decreased in the ovaries. Upregulation of miR-2 or novel-miR-109 also resulted in reduced fecundity and ovary defects similar to those caused by *DcKr-h1* silencing. Moreover, feeding antagomir-2 or antagomir-109 partially rescued the defective phenotypes caused by *DcKr-h1* silencing in both model systems, and miR-2 and novel-miR-109 were repressed by juvenile hormone (JH) and regulated the genes associated with egg development. This study shows a conserved regulatory mechanism, whereby JH suppresses the expression of miR-2 and novel-miR-109 which, together with JH-induced transcription of *DcKr-h1*, increases female fecundity induced by both symbiotic bacteria and pathogenic fungi.

**IMPORTANCE:**

Infection with pathogens can increase the fecundity and other fitness-related traits of insect vectors for their own advantage. Our previous research has reported that *DcKr-h1* plays a critical role in the increase in fecundity of *Diaphorina citri* induced by the bacterium, “Candidatus Liberibacter asiaticus” (*C*Las) and the fungus, *Cordyceps fumosorosea* (*Cf*). However, the posttranscriptional regulation of this process remains poorly understood. Given the significance of miRNAs in gene regulation, we delved into their roles in shaping phenotypes and their underlying molecular mechanisms. Our results indicated that two miRNAs, miR-2 and novel-miR-109, jointly inhibited *DcKr-h1* expression by binding to its 3′ untranslated region (UTR). In both *D. citri*-*C*Las and *D. citri*-*Cf* interactions, the increased juvenile hormone (JH) titer and reduced abundance of miR-2 and novel-miR-109 ensure high levels of *DcKr-h1* expression, consequently stimulating ovarian development and enhancing fecundity. These observations provide evidence that miR-2 and miR-109 are crucial players in the JH-dependent increase in fecundity in psyllids induced by infection with different pathogens.

## INTRODUCTION

Numerous devastating microbes, including viruses, microsporidia, bacteria, and fungi, are transmitted by insect vectors (1–4). The evolutionary dynamic of insect vector–pathogen relationships has long captivated researchers in the field of molecular ecology and evolutionary biology (5–8). A deep comprehensive understanding of these interactions is pivotal for understanding the epidemiology of plant pathogens and devising new strategies for managing both insect vectors and the pathogens they carry. During recent decades, extensive studies have been undertaken to decipher the molecular mechanisms of insect vector–pathogen interactions (6, 9, 10). Pathogen infections can trigger alterations in vector morphology, physiology, behavior, and reproduction, with the latter facilitating transmission of pathogens (3, 4). However, the molecular mechanisms behind the changes in vector reproduction induced by different pathogens remain largely unexplored.

MicroRNAs (miRNAs) are a family of small non-coding RNAs that typically exert negative regulation on gene expression by binding to the 3′ untranslated region (UTR) of target mRNAs, thereby either inhibiting translation or promoting mRNA degradation (11, 12). miRNAs play important roles in female reproduction (13). For example, in *Aedes aegypti* (Linnaeus in Hasselquist) (Diptera: Culicidae), miR-309, miR-275, and miR-8 are essential for different reproductive processes (14–16). In *Bombyx mori* L. (Lepidoptera: Bombycidae), miR-2739 and miR-167 coordinately regulate the expression of the vitellogenin receptor (VgR) during ovarian development (17). However, researches on miRNAs involved in reproductive regulation have primarily focused on model insects such as *Drosophila melanogaster* Meigen (Diptera: Drosophilidae), *A. aegypti*, and *B. mori* (13). Further exploration involving less-studied insects is warranted to elucidate the functions of miRNAs governing female insect reproduction. In addition, the pivotal roles of miRNAs in insect vector–pathogen interactions are increasingly recognized. For examples, in the host-virus interaction between *Helicoverpa armigera* (Hübner) (Lepidoptera: Noctuidae) and a nucleopolyhedrovirus (HaSNPV), miR-8 and miR-429 target the *Broad isoform Z2* gene, which modulates the hormonal regulation of virus-mediated climbing behavior (18). In the host-bacterium interaction between “*Candidatus* Liberibacter asiaticus” Jagoueix et al*.* (Alphaproteobacteria) (*C*Las) and *Diaphorina citri* Kuwayama (Hemiptera: Psyllidae), the bacterium hijacks host miR-275 that targets the gene encoding VgR. This manipulation boosts the fecundity of its vector, while simultaneously increasing the replication of *C*Las in ovaries, suggesting a mutualistic interaction between *D. citri* and *C*Las (19). Nonetheless, the broad functions of miRNAs in vector–pathogen interactions, especially on vector reproduction, require further investigation.

*D. citri* is one of the most destructive pests affecting various citrus species and hybrids worldwide (20) due to its role in transmitting *C*Las, the phloem-restricted bacterium associated with the severe Asian form of huanglongbing (21). Notably, *D. citri* infected with *C*Las exhibit higher fecundity levels compared to their *C*Las-negative counterparts (22–24). In insects, the regulation of reproduction is chiefly governed by juvenile hormone (JH) (13). Following *C*Las infection, the upregulation of the JH signaling pathway occurs, thereby increasing the fecundity of *D. citri* through the action of the JH receptor encoded by *methoprene-tolerant* (*DcMet*) and the downstream transcription factor *Krappel homolog 1* (*DcKr-h1*) (19). Interestingly, exposing fifth-instar nymphs of *D. citri* to low concentrations of *Cordyceps fumosorosea* (*Cf*) (Wize) (Hypocreales: Cordycipitaceae) also leads to a significant increase in fecundity compared to uninfected individuals, with changes in *DcMet* and *DcKr-h1* contributing to the fecundity alterations caused by this pathogen (25). These findings collectively suggest a functional conservation of the JH-Met-Kr-h1 signaling pathway in regulating *D. citri*-pathogen interactions.

*Kr-h1* serves as an early JH-responsive gene downstream of the JH receptor, playing an indispensable role in insect metamorphosis and reproduction (26, 27). The JH receptor complex binds to JH response elements in the *Kr-h1* promoter, directly regulating its transcription (26, 28, 29). To date, research on miRNAs targeting *Kr-h1* has been limited to a few insect species. For example, in *Blattella germanica* L. (Blattodea: Ectobiidae), the members of the miR-2 family (such as miR-2, miR-13a, and miR-13b) are upregulated during the final nymphal instar, coordinately suppressing *Kr-h1* expression and playing a crucial role in initiating metamorphosis (30). In *Locusta migratoria* (L.) (Orthroptera: Acrididae), miR-278 and let-7 jointly regulate metamorphosis and oogenesis by targeting *Kr-h1* (31). However, there is a lack of reports on miRNAs targeting *Kr-h1* in the context of vector–pathogen interactions. *C*Las, a typical propagative-circulative bacterium, and *C. fumosorosea*, an entomopathogenic fungus used for controlling *D. citri* and other insects, both lead to increased *Kr-h1* transcription following infection, playing a conserved role in the enhancing psyllid fecundity caused by pathogen infection (19, 25). However, further investigations are warranted to determine whether the function of the miRNAs targeting *Kr-h1* is conserved in these two interactions. Therefore, in the present study, we used two interaction systems of *D. citri-C*Las and *D. citri-Cf* to investigate the possible conserved functions of miRNAs targeting *Kr-h1* in enhancing fecundity of *D. citri*.

## RESULTS

### miR-2 and novel-miR-109 target *DcKr-h1*

**FIG 1:**
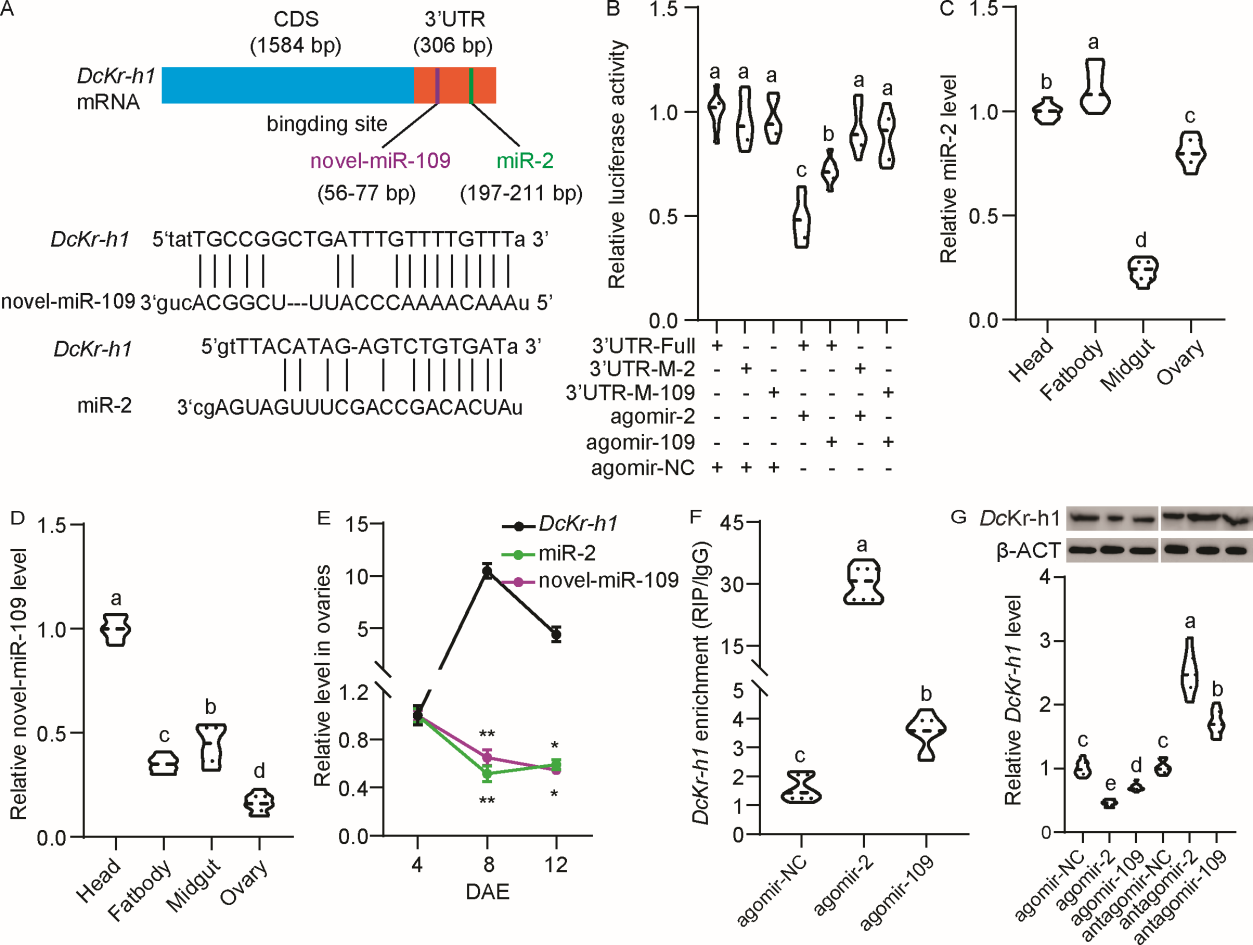
Identification of miR-2 and novel-miR-109 potentially targeting *DcKr-h1*. (A) Predicted binding sites of miR-2 and novel-miR-109 within the *DcKr-h1* 3′-UTR using miRanda and RNAhybrid. The rectangular light blue and red boxes represent the CDS region and 3′UTR of *DcKr-h1*, respectively, while the blue and green vertical bars represent the binding sites of novel-miR-109 and miR-2, respectively. (B) Dual-luciferase reporter assays using HEK293T cells co-transfected with miRNA agomirs and recombinant pmirGLO vectors containing either the wild-type *DcKr-h1-*3′UTR or mutated *DcKr-h1-*3′ UTR. 3′UTR-M-2: mutation in the binding sites complementary to the seed sequences of miR-2; 3′UTR-M-109: mutations in the biding sites complementary to the seed sequences of miR-109. (C) Tissue-specific expression of miR-2 in the head, fat body, midgut, and ovary of adults female *D. citri* at 7 DAE. (D) Tissue-specific expression of novel-miR-109 in the head, fat body, midgut, and ovary of adults female *D. citri* at 7 DAE. (E) Comparison of temporal expression patterns of miR-2, novel-miR-109, and *DcKr-h1* in the ovaries of adults female *D. citri* at 4, 8, and 12 DAE. (F) *In vivo* expression analysis of miR-2 and novel-miR-109 targeting *DcKr-h1* using an RIP assay. (G) Effects of miRNA agomir and antagomir treatments at 48 h on *DcKr-h1* mRNA and protein levels in the ovaries of adults female *D. citri*. Data represent three biological replicates with three technical replicates and are shown as means ± standard errors (SEs). Different letters above the hourglasses indicate significant group differences determined by one-way ANOVA followed by Tukey’s HSD test (*P* < 0.05). The significant between differences are indicated by asterisks were determined by student’s *t*-tests (*P < 0.05, **P < 0.01).

Utilizing miRanda and RNAhybrid software tools, three miRNAs were predicted to target *Dckr-h1*: miR-2, novel-miR-109, and miR-171 (Fig. 1A; Fig. S1A). To validate the predicted binding interaction of these miRNAs, a luciferase reporter plasmid containing *DcKr-h1* 3′UTR and miRNA agomirs were co-transfected into HEK293T cells, followed by luciferase activity assayed. Results indicated that miR-2 and novel-miR-109 reduced luciferase activity by 52% and 29%, respectively, whereas agomir-171 showed no significant effect on luciferase activity compared to negative control agomirs (Fig. S1B). Notably, mutation of the miR-2 or miR-109 binding sites within the *DcKr-h1* 3′UTR led to the restoration of luciferase reporter activities (Fig. 1B). Tissue-specific expression profiling showed that miR-2 exhibited the highest expression in the fat bodies, followed by the head, ovaries, and midgut (Fig. 1C); while novel-miR-109 displayed peak expression in the head, followed by the midgut, fat bodies, and ovaries (Fig. 1D). In addition, miR-2 and novel-miR-109 exhibited inverse expression patterns with *DcKr-h1* in the ovaries (Fig. 1E). In an RNA immunoprecipitation (RIP) assay, psyllids fed with agomir-2 and agomir-109 showed a significant increase in the levels of these miRNAs compared to negative controls (Fig. S2). Moreover, the abundances of *DcKr-h1* mRNA were upregulated by 19.1-fold and 2.3-fold up-regulated when agomir-2 and agomir-109 were supplied in the Ago-1 antibody-mediated RNA complex, compared to an IgG control (Fig. 1F). Treatment with agomir-2 or -109 resulted in elevated expression levels of these miRNAs (Fig. S3), accompanied by reduced *DcKr-h1* expression at both mRNA and protein levels. Conversely, treatment with antagomir-2 or -109 led to increased levels of *DcKr-h1* mRNA and protein expression (Fig. 1G), suggesting a regulatory role for miR-2 and miR-109 in targeting the *DcKr-h1* 3′UTR to modulate its expression.

### miR-2 and novel-miR-109 target *DcKr-h1* and are involved in the increased fecundity of *D. citri* induced by *C*Las

**FIG 2:**
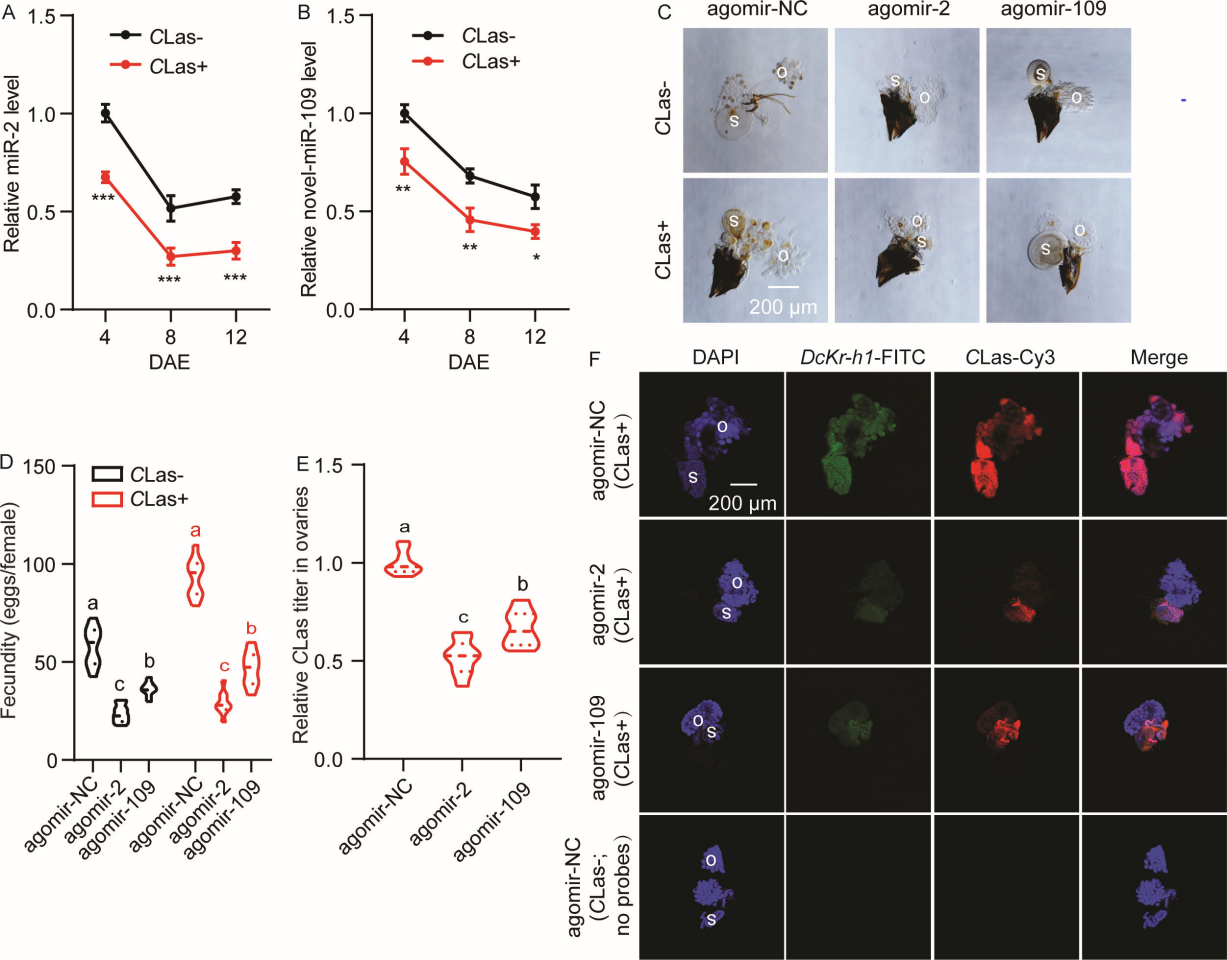
miR-2 and miR-109 participated in the increase in fecundity of *D. citri* induced by *C*Las infection. (A) Comparison of the temporal expression patterns of miR-2 in the ovaries of *C*Las- and *C*Las+ females. (B) Comparison of the temporal expression patterns of novel-miR-109 in the ovaries of *C*Las- and *C*Las+ females. (C) Morphological analysis of ovaries from *C*Las- and *C*Las+ females treated with agomir-2 or agomir-109 for 48 h. Scale bar = 200 µm. (D) Comparison of the fecundity between *C*Las- and *C*Las+ females treated with agomir-2 or agomir-109. (E) *C*Las titer in the ovaries of *C*Las+ females treated with agomir-2 or agomir-109 for 48 h. (F) Representative confocal images of *DcKr-h1* and *C*Las in the reproductive system of *C*Las+ females fed with agomir-2 or agomir-109 for 48 h. Scale bar is 200 µm. 4',6-Diamidino-2-phenylindole (DAPI): cell nuclei stained with DAPI and visualized in blue. *DcKr-h1*-fluorescein isothiocyanate (FITC): the *DcKr-h1* signal is visualized in green by staining with FITC. *C*Las-Cy3: the *C*Las signal is visualized in red by staining with Cy3. Merge: merged imaging of co-localization of cell nuclei, *DcDcKr-h1*, and *C*Las. Data represent three biological replicates with three technical replicates and are shown as means ± SEs. Different letters above the hourglasses indicate significant group differences determined by one-way ANOVA followed by Tukey’s HSD tests (*P* < 0.05). Significant differences indicated by asterisks were determined by student’s *t*-tests (*P < 0.05, **P < 0.01, ***P < 0.001) for A and B. o, ovary; s, spermathecae.

To investigate the roles of miR-2 and novel-miR-109 in the interaction between *D. citri* and *C*Las, the developmental patterns of miR-2 and miR-109 in the ovaries of *C*Las-negative (*C*Las-) and *C*Las-positive (*C*Las+) psyllids were examined. Our analysis showed a decline in the transcript levels of miR-2 and novel-miR-109 over the assessment period, with both miRNAs exhibiting lower expression in *C*Las+ individuals compared to *C*Las-psyllids (Fig. 2A and B). The time-dependent expression patterns of miR-2 and miR 109 could be linked to the expression of the target gene *DcKr-h1* and its role in regulating female reproduction. The decrease in their expression at 8 DAE may be attributed to the majority of eggs maturity and the upregulation of *DcKr-h1*. Subsequent treatment with miRNA agomirs resulted in a deceleration of ovarian development in both *C*Las- and *C*Las+ psyllids, leading to a reduction in ovarian size (Fig. 2C). Notably, compared with the controls, when *C*Las- and *C*Las+ psyllids were fed with agomir-2 and agomir-109, the preoviposition periods were prolonged (from 11.0 d to 13.7 d and 12.8 d in *C*Las- psyllids, and from 9.6 d to 12.3 d and 11.7 d in *C*Las+ psyllids), and the oviposition periods were markedly shortened (from 10.8 d to 5.7 d and 7.9 d in *C*Las- psyllids, and from 13.8 d to 5.6 d and 7.4 d in *C*Las+ psyllids) (Table 1). The fecundity of *C*Las- and *C*Las+ females treated with agomir-2 or agomir-109 was significantly decreased (from 57.6 to 23.7 eggs and 36.12 eggs in *C*Las- psyllids and from 92.9 to 26.0 eggs and 46.4 eggs in *C*Las+ psyllids) (Fig. 2D). Furthermore, following the administration of agomir-2 and agomir-109 to *C*Las+ females, qRT-PCR analysis showed a significant reduction in relative *C*Las titers in the ovaries (Fig. 2E), while FISH assays revealed a substantial decrease in both *C*Las and *DcKr-h1* signals within the ovaries (Fig. 2F).

**TABLE 1:** Different treatments on the preoviposition period and oviposition period of *D*. *citri* females^*a*^

Experiments	Treatments	Preoviposition period	Oviposition period
miRNA agomirs (*C*Las-)	agomir-NC	11.0 ± 0.3 a	10.8 ± 0.4 a
	agomir-2	13.7 ± 0.4 b	5.7 ± 0.4 c
	agomir-109	12.8 ± 0.2 b	7.9 ± 0.4 b
miRNA agomirs (*C*Las+)	agomir-NC	9.6 ± 0.1 a	13.8 ± 0.6 a
	agomir-2	12.3 ± 0.3 b	5.7 ± 0.5 b
	agomir-109	11.7 ± 0.2 b	7.4 ± 0.4 b
miRNA antagomirs Rescue (*C*Las+)	ds*GFP*	9.8 ± 0.2 b	13.1 ± 0.1 a
	ds*DcKr-h1*	12.7 ± 0.3 a	5.0 ± 0.4 c
	agomir-NC	10.1 ± 0.2 b	13.7 ± 0.5 a
	agomir-2	12.4 ± 0.3 a	5.4 ± 0.4 c
	agomir-109	12.7 ± 0.3 a	7.1 ± 0.4 bc
	antagomir-NC	10.0 ± 0.2 b	12.7 ± 0.7 a
	ds*DcKr-h1*+antagomir-2	11.8 ± 0.3 a	7.6 ± 0.4 b
	ds*DcKr-h1+*antagomir-109	12.2 ± 0.3 a	6.5 ± 0.4 bc
miRNA agomirs (CK)	agomir-NC	11.0 ± 0.3 b	10.8 ± 0.3 a
	agomir-2	12.9 ± 0.5 a	6.6 ± 0.3 b
	agomir-109	13.4 ± 0.4 d	5.8 ± 0.2 b
miRNA agomirs (*Cf+*)	agomir-NC	8.4 ± 0.2 b	7.6 ± 0.5 a
	agomir-2	12.3 ± 0.4 a	4.3 ± 0.2 b
	agomir-109	12.9 ± 0.3 a	5.3 ± 0.3 b
miRNA antagomirs rescue (*Cf+*)	ds*GFP*	8.6 ± 0.1 b	7.9 ± 0.5 a
	ds*DcKr-h1*	10.0 ± 0.3 a	3.8 ± 0.3 b
	agomir-NC	8.5 ± 0.2 b	8.6 ± 0.4 a
	agomir-2	9.8 ± 0.3 a	4.7 ± 0.4 b
	agomir-109	10.0 ± 0.4 a	5.0 ± 0.7 b
	antagomir-NC	8.3 ± 0.1 b	8.1 ± 0.5 a
	ds*DcKr-h1*+antagomir-2	9.8 ± 0.3 a	5.5 ± 0.2 b
	ds*DcKr-h1*+antagomir-109	10.1 ± 0.3 a	4.8 ± 0.5 b

^*a*^ Different letters in the same experiment indicate significant differences determined by one-way ANOVA followed by Tukey’s HSD test (*P* < 0.05).

### miR-2 and novel-miR-109 target *DcKr-h1* and are involved in the increased fecundity of *D. citri* induced by *C. fumosorosea* (*Cf*)

**FIG 3:**
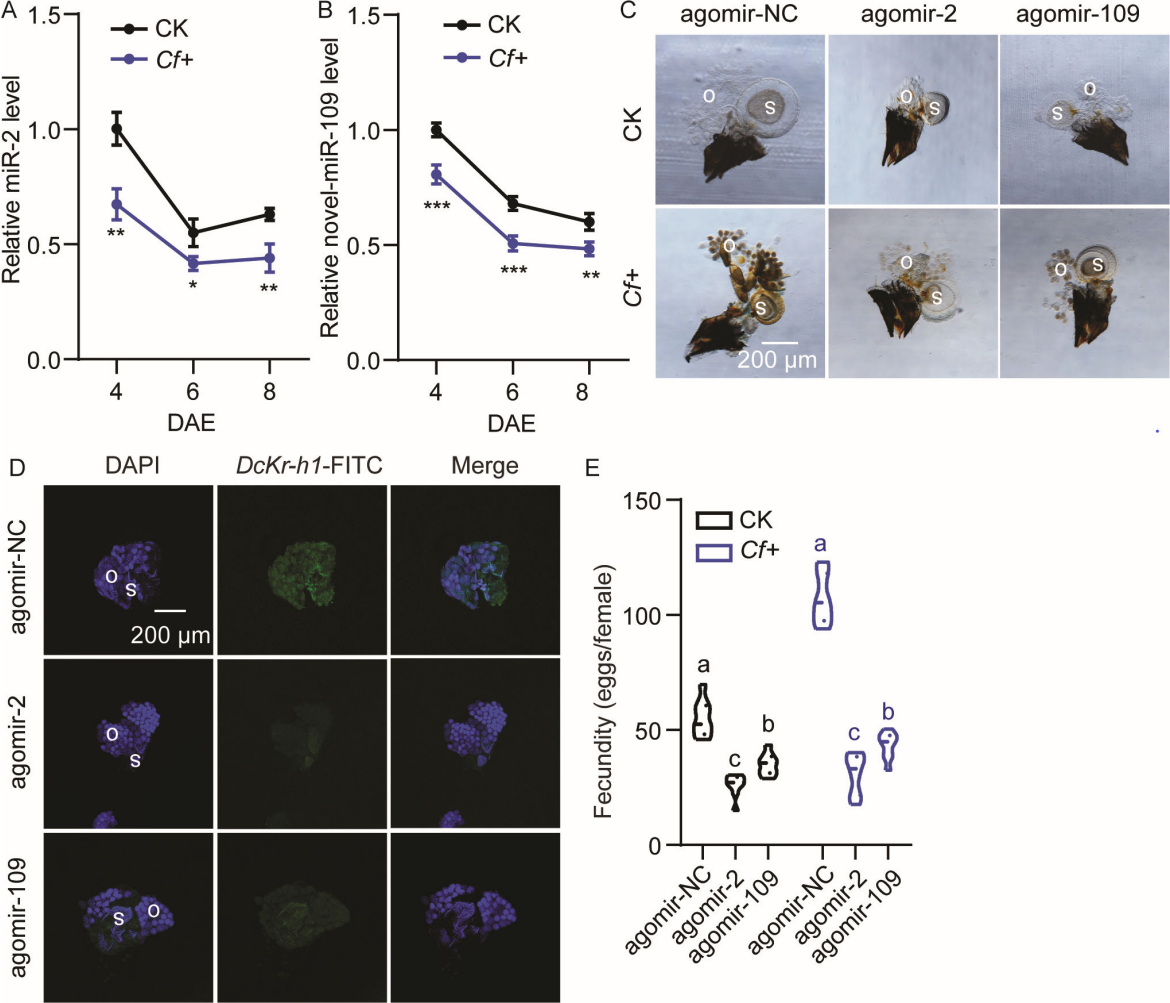
miR-2 and miR-109 participate in the increased fecundity of *D. citri* induced by *Cf.* (A) Comparison of temporal expression pattern of miR-2 in the ovaries of the control (CK: 0.05% Tween-80) and *Cf*+ females. (B) Comparison of temporal expression pattern of novel-miR-109 in the ovaries of the control and *Cf*+ females. (C) Ovary phenotypes of CK and *Cf*+ females fed with agomir-2 or agomir-109 for 48 h. Scale bar = 200 µm. (D) The *DcKr-h1* signal in *Cf*+ females fed with agomir-2 or agomir-109 for 48 h. The signals for DAPI and *DcKr-h1*-FITC are same as described in Fig. 2. (E) Comparison of the fecundity between control and *Cf*+ females fed with agomir-2 or agomir-109. Data represent three biological replicates with three technical replicates and are shown as means ± SEs. Different letters above the hourglasses indicate significant differences determined by one-way ANOVA followed by Tukey’s HSD tests (*P* < 0.05). Significant differences determined by Student’s *t*-tests are indicated by asterisks (*P < 0.05, **P < 0.01, ***P < 0.001). o, ovary; s, spermathecae.

To investigate the roles of miR-2 and novel-miR-109 in the interaction between *D. citri* and *Cf*, the developmental patterns of these miRNAs in the ovaries of *Cf*-treated (*Cf*+) psyllids and the controls were assayed. Our findings revealed a decrease in the transcript levels of miR-2 and miR-109 over the assessment period, with lower levels observed in *Cf+* psyllids than in the controls (Fig. 3A and B). Subsequent treatment with miRNA agomirs resulted in a slowdown of ovarian development in both control and *cf-*infected psyllids, leading to reduced ovarian size (Fig. 3C). FISH assays following miRNA agomirs feeding demonstrated a significant decrease in the *DcKr-h1* signal in the ovaries (Fig. 3D). Moreover, the preoviposition period was prolonged (from 11.0 to 13.0 d and 13.4 d in the control psyllids, from 8.4 to 12.3 d and 12.9 d in *Cf*+ psyllids), while the oviposition period was markedly shortened (from 10.8 to 6.6 d and 5.8 d in the control psyllids, from 7.6 to 4.3 d and 5.3 d in *Cf*+ psyllids), and the fecundity significantly decreased (from 55.2 to 26.0 eggs and 35.1 eggs in the control psyllids, from 106.9 to 30.8 eggs and 43.4 eggs in *Cf+* psyllids) compared with the controls (Fig. 3E; Table 1).

### miRNA antagomir treatments partially rescue ds*DcKr-h1* phenotypes

**FIG 4:**
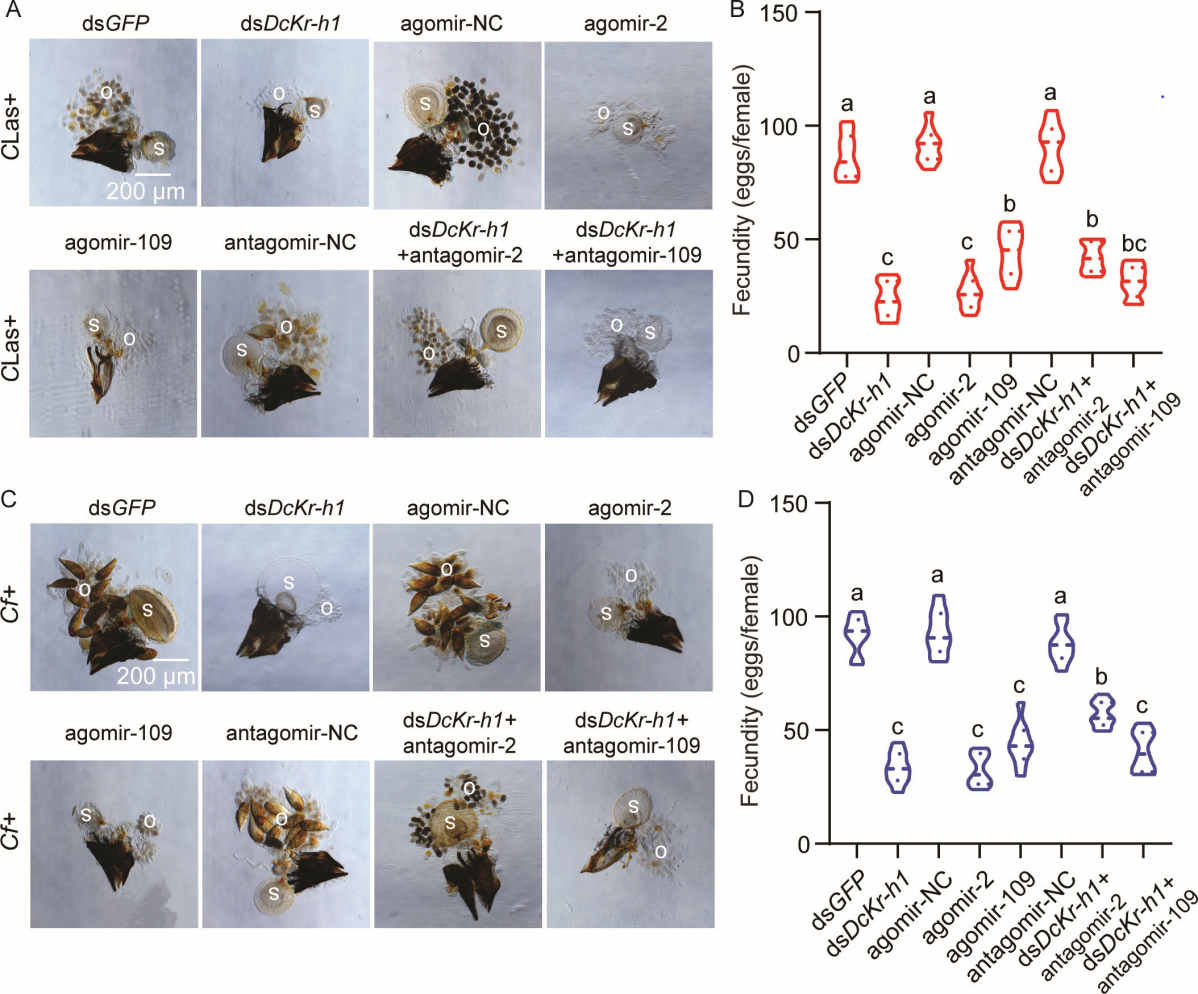
Feeding with antagomirs partially rescues the ovarian defects induced by feeding with ds*DcKr-h1* in the *D. citri-C*Las and *D. citri-Cf* interactions. (A and B) Ovary phenotypes and fecundity of *C*Las+ females fed with ds*DcKr-h1,* agomir treatments, and antagomir rescues. Scale bar = 200 µm. (C and D) Ovary phenotypes and fecundity of *Cf*+ females fed with ds*DcKr-h1,* agomir treatments, and antagomir rescues. Scale bar = 200 µm. o: ovary, s: spermathecae. Data represent three biological replicates with three technical replicates and are shown as means ± SEs. Different letters above the hourglasses indicate significant differences determined by one-way ANOVA followed by Tukey’s honest significant difference tests (*P* < 0.05).

The perturbation in ovarian development and fecundity caused by treatment with miR-2 or novel-miR-109 agomirs resembled that resulting from *DcKr-h1* RNAi reported by Nian et al. (19) and Wu et al. (25). Feeding with antagomir-2 partially rescued ovarian development in *C*Las + psyllids treated with ds*DcKr-h1*, including the size of the ovaries (Fig. 4A), *C*Las titer and the expression of *DcKr-h1* in ovaries (Fig. S4A and B), and fecundity (Fig. 4B), as well as the preoviposition periods and oviposition periods (Table 1). However, feeding with antagomir-109 slightly rescued ds*DcKr-h1* phenotypes, including the size of the ovaries, *C*Las titer, the expression of *DcKr-h1* in ovaries, oviposition period, and fecundity, although the differences were not significant (Fig. 4A and B; Fig. S4A and B; Table 1). Similar results were observed when *Cf*+ psyllids were fed with antagomir treatments. Only antagomir-2 treatment partially rescued the size of the ovaries, the fecundity of *dsDcKr-h1* (Fig. 4C and D), the expression of *DcKr-h1* in ovaries (Fig. S4C), the preoviposition periods, and oviposition periods (Table 1). Taken together, these findings indicate that the targeting of *DcKr-h1* by miR-2 and novel-miR-109 plays a crucial role in the *D. citri-C*Las and *D. citri-Cf* interactions that results in increased fecundity.

### The JH signaling pathway is regulated by miR-2 and novel-miR-109 in both the *D. citri-C*Las and *D. citri-Cf* interactions

**FIG 5:**
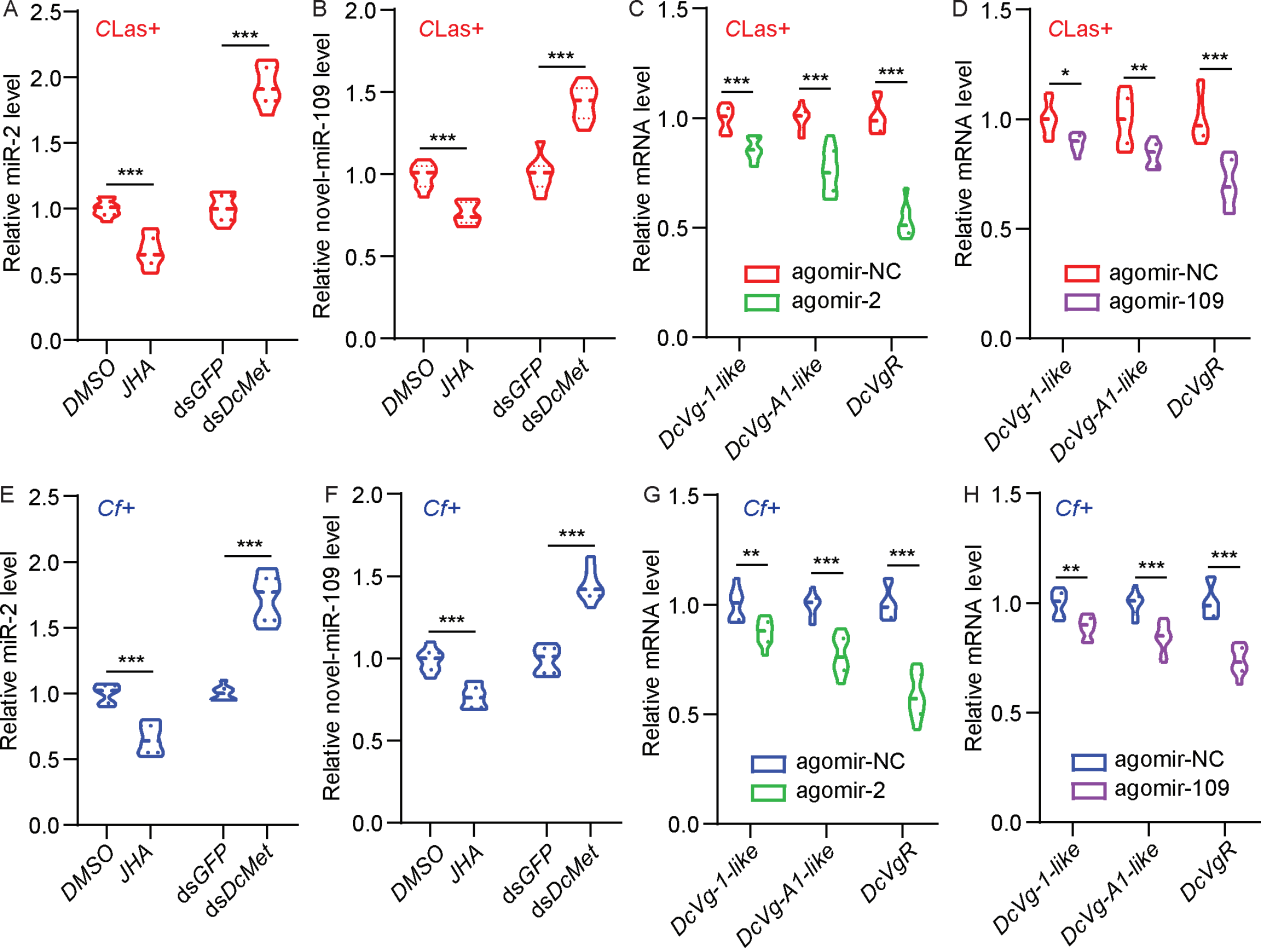
The JH signaling pathway is regulated by miR-2 and novel-miR-109 in both the *D. citri-C*Las and *D. citri-Cf* interactions. (A) Effects of methoprene and ds*DcMet* treatments on miR-2 levels in the ovaries of *C*Las+ psyllids. (B) Effects of methoprene and ds*DcMet* treatments on novel-miR-109 level in the ovaries of *C*Las+ psyllids. (C) Influence of agomir-2 treatment on mRNA levels of *DcVg-1-like*, *DcVg-A1-like*, and *DcVgR* in the ovaries of *C*Las+ females. (D) Impacts of agomir-109 treatment on mRNA levels of *DcVg-1-like*, *DcVg-A1-like*, and *DcVgR* in the ovaries of *C*Las+ females. (E) Effects of methoprene and ds*DcMet* treatments on miR-2 level in the ovaries of *Cf*+ females. (F) Influence of methoprene and ds*DcMet* treatments on miR-109 level in the ovaries of *Cf+* females. (G) Impacts of agomir-2 treatment on mRNA levels of *DcVg-1-like*, *DcVg-A1-like*, and *DcVgR* in the ovaries of *Cf+* females. (H) Influence of agomir-109 treatment on mRNA levels of *DcVg-1-like*, *DcVg-A1-like*, and *DcVgR* in the ovaries of *Cf+* females. Data represent three biological replicates with three technical replicates and are shown as means ± SEs. Significant differences determined by student’s *t*-tests are indicated by asterisks (*P < 0.05, **P < 0.01, ***P < 0.001).

Previous studies showed a notable increase in JH titer in the ovaries of *C*Las +or *Cf* +psyllids during ovarian development compared to the controls (19, 25). Subsequently, we administered the JH analog, methoprene, to *C*Las +or *Cf+* females for 24 h, resulting in a 32.6% and 23.7% decrease in the mRNA level of miR-2 and miR-109 in the ovaries, respectively (Fig. 5A and B). Knockdown of *DcMet* led to a significant increase in the expression of miR-2 and miR-109 in the ovaries of both *C*Las +and *Cf* +psyllids (Fig. 5A and B). Furthermore, after feeding with agomir-2 or agomir-109 for 48 h, the expression levels of *DcVg1-like*, *DcVgA1-like*, and *DcV*gR, the three important downstream genes of the JH signaling pathway related to ovarian development, were significantly reduced in the ovaries of *C*Las +females (Fig. 5C and D). Similar results were observed in *Cf+* females upon treatment with agomir-2 or agomir-109 (Fig. 5E through H). These results suggest that JH signaling pathway is regulated by miR-2 and novel-miR-109 and plays a role in the increased fecundity of *D. citri* induced by *C*Las and *Cf* infection.

**FIG 6:**
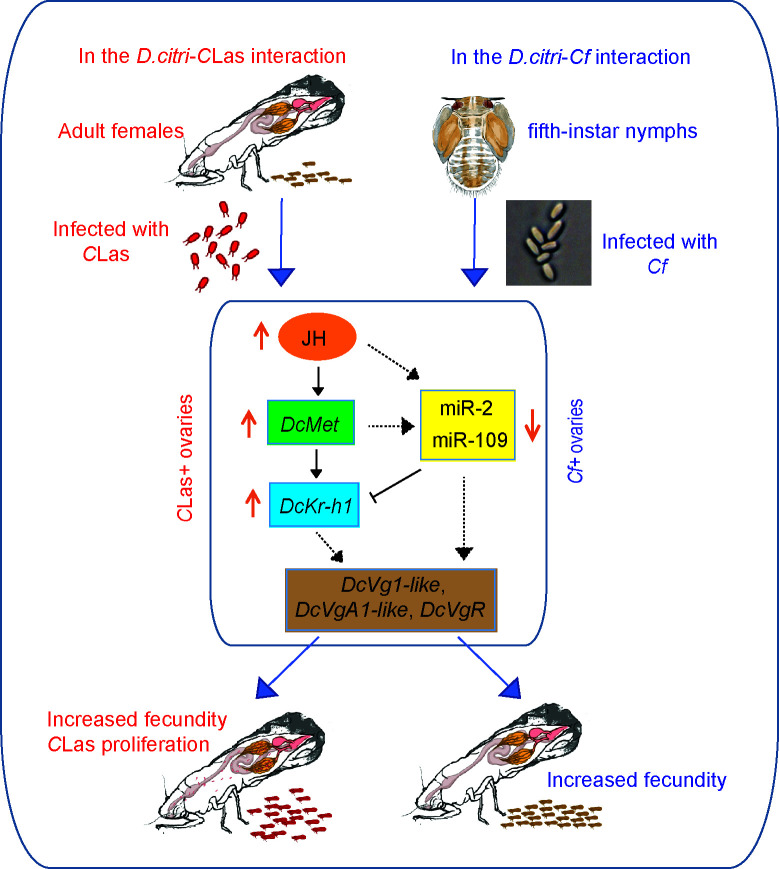
The conservative mechanisms of miR-2 and novel miR-109 in the fecundity improvement of *D. citri* induced by *C*Las and *Cf*. In *C*Las+ ovaries, *C*Las upregulates the JH-Met-Kr-h1 signaling pathway and downregulates miR-2 and novel-miR-109 to activate JH-dependent vitellogenesis, thereby improving the fecundity of *C*Las+ females. In the ovaries of *Cf*+ psyllids, fungal infection also upregulated the JH-Met-Kr-h1 signaling pathways and downregulated miR-2 and novel-miR-109 to activate JH-dependent vitellogenesis, thereby increasing the fecundity of infected females.

## DISCUSSION

Pathogen infection can confer advantages by increasing the reproductive fitness and other fitness-related traits in insect vectors. Consequently, an increasing number of studies have focused on the effects of insect–pathogen interactions on insect reproduction. For example, in the realm of insect–virus interactions, Jiu et al. (32) reported that mutualism between vectors and viruses increased the fecundity and longevity of the invasive B biotype *Bemisia tabaci* (Gennadius) (Hemiptera: Aleyrodidae), resulting in population expansion. However, the indigenous Q biotype appeared to lack such mutualism benefits. This mutualistic interaction likely played a pivotal role in the successful invasion of the B biotype, the displacement of the indigenous Q biotype, and the spread of viral diseases associated with this vector. Moeini et al. (33) discovered that the acquisition of Maize Iranian mosaic virus (MIMV) by *Laodelphax striatellus* Fallén (Delphacidae: Hemiptera) resulted in increased female fecundity, prolonged nymphal stage duration, extended adult longevity, and enhanced survival time. These findings suggest that MIMV has the potential to enhance the ecological fitness of its vector, potentially facilitating the spread of the virus. In the context of the vector–bacterium interaction between *D. citri* and *C*Las, infection has been shown to accelerate development and increase reproductive fitness, ultimately resulting in population growth (22). Singh and Linksvayer (34) demonstrated that colonies of *Wolbachia*-infected pharaoh ants (*Monomorium pharaonis* (L.) (Hymenoptera; Formicidae)) exhibited enhanced colony-level growth and earlier reproduction, and a shorter colony life cycle compared to uninfected colonies. The investigation of how insect–pathogen interactions influence insect reproduction has been a prominent area of research in ecology, entomology, and microbiology (3, 4). Nevertheless, our understanding of the molecular mechanisms behind the changes in vector reproduction induced by different pathogens remains limited.

Kr-h1, a zinc-finger transcription factor, is a key player in the JH signaling pathway. The JH receptor complex interacts with JH response elements within the *Kr-h1* promoter, directly influencing its transcription (26, 28, 29). During larval stages, Kr-h1 mediates the anti-metamorphic action of JH in both hemimetabolous and holometabolous insets (35). However, the role of Kr-h1 in female reproduction appears to exhibit species-specific variations. For example, Kr-h1 promotes vitellogenesis, ovarian development, and oocyte maturation in *L. migratoria* (26), whereas in *Cimex lectularius* L. (Hemiptera: Cimicidae), the knockdown of Kr-h1 does not reduce egg production but severely decreases hatching ability (36). Despite extensive studies aimed at elucidating the functions of *Kr-h1* at the transcriptional level, only a few miRNAs have been demonstrated to bind to the 3’UTR or coding sequence (CDS) of *Kr-h1*, such as miR-278, let-7, and miR-2 family members (miR-2, miR-13a, and miR-13b) (30, 31). Through a combination of *in vitro* and *in vivo* experiments, the current study found that miR-2 and novel-miR-109 target the 3’UTR of *DcKr-h1*. Notably, miR-2 and novel-miR-109 exhibited inverse expression patterns relative to those of *DcKr-h1*. Treatment with either miR-2 or novel-miR-109 agomirs and antagomirs resulted in significant decreases and increases, respectively, in *DcKr-h1* mRNA and protein level. miR-2 has previously been reported to downregulate *Kr-h1* expression via binding to its 3’UTR (30), thereby expanding our understanding of Kr-h1 regulation at the posttranscriptional level. Song et al. (31) reported that miRNAs, let-7 and miR-278, regulate metamorphosis and oogenesis in *L. migratoria* by targeting the *Kr-h1* CDS. Notably, no miR-2 binding site was predicted in the locust *Kr-h1* mRNA sequence, suggesting the diversity of miRNAs and their targets within this gene across different insect species.

In host cells, a multitude of miRNAs have been identified to alter gene expression in response to pathogen infection. For instance, the Zika virus has been shown to influence the expression of 17 miRNAs and induce RNA interference responses in *A. aegypti* (37). Host miRNAs play a crucial role in mounting defense responses against pathogen infections (38, 39). Wang et al. (40) demonstrated that *Anopheles stephensi* Liston (Diptera: Culicidae) and *B. mori* (L.) (Lepidoptera: Bombycidae) transfer miRNAs to mycelium of the pathogenic fungus *Beauveria bassiana* (Bals.-Criv.) Vuill. (Hypocreales: Cordycipitaceae). The translocated miRNAs then suppress the expression of fungal genes crucial for virulence. Notably, the expression of the miRNA, bantam, was found to increase in Sf9 cells and *Spodoptera litura* (Fabricius) (Lepidoptera: Noctuidae) larvae following infection with Autographa Californica Multiple Nucleopolyhedrovirus (41). Manipulation of bantam levels resulted in altered virus DNA replication and abnormal larval growth, underscoring the inricate interplay between host miRNAs and pathogen dynamics. Pathogens can also hijack host miRNAs to change host activities including escaping the host immune response and facilitate their replication (19, 31). These findings suggested that miRNAs may either function as a host antiviral response or are manipulated by microbes to favor their replication within the host, highlighting their important functions in insect–microbe interactions. Despite the identification of several miRNAs involved in vector–pathogen interactions, limited research has focused on the conserved functions of miRNAs in vector–fungus and vector–bacterium interactions.

In the current study, miR-2 and novel-miR-109, targeting *DcKr-h1*, were involved in the increases in fecundity of *D. citri* induced by different pathogens. In the *D. citri-C*Las interaction, the expression levels of miR-2 and novel-miR-109 in the ovaries of *C*Las +psyllids were lower compared to *C*Las- individuals. Overexpression of miR-2 and novel-miR-109 not only reduced fecundity but also decreased *DcKr-h1* levels and *C*Las titers in the ovaries, resulting in phenotypes similar to those caused by *DcKr-h1* silencing (19). In addition, defective reproductive phenotypes and decreased *C*Las titer caused by ds*DcKr-h1* treatment could be partially rescued by treatment with antagomir-2. Similar reproductive defects were found in the *D. citri-Cf* interaction; following treatment with *Cf*, the expression levels of miR-2 and novel-miR-109 in the ovaries of females significantly decreased compared to controls. Application of miR-2 and novel-miR-109 agomirs led to decreased *DcKr-h1* levels in the ovaries, reducing fecundity and mirroring the effects of *DcKr-h1* RNAi, because both ds*DcKr-h1* and miRNA agomir treatments resulted in a reduction in the expression of *DcKr-h1*; the defective phenotype could be partially restored by treatment with antagomir-2 and antagomir-109, although the rescue effect of antagomir-109 treatment was not statistically significant. The concept behind antagomirs is to disrupt the binding of miRNAs to their target gene mRNA by competitively binding with mature miRNAs in the organism, thereby impeding the activity of the miRNAs. However, there are two possible reasons why antagomir-109 could only slightly rescue the defective phenotypes induced by ds*DcKr-h1*. Basically, this could be attributed to its limited abundance in the ovaries. Next, it is plausible that these two miRNAs affect the transcription of the target gene by regulating other target genes, given that a single miRNA typically targets multiple genes, and conversely, multiple miRNAs can regulate a single gene (11, 42). In a previous study (19), we demonstrated that *Kr-h1* knockdown in *C*Las +female psyllids caused a substantial reduction in *DcVgR* expression and slowed ovarian development. In this study, both miR-2 and novel-miR-109 agomir treatments significantly reduced the expression levels of egg development-related genes, *DcVg-1-like*, *DcVg-A1-like*, and *DcVgR*, resulting in inhibited ovarian growth. Regardless of whether infected with a bacterium (*C*Las) or with a fungus (*Cf*), the fecundity of *D. citri* was significantly increased, accompanied by elevated JH titers and the expression of *DcMet* and *DcKr-h1* in female adults ovaries. Moreover, our study found that the expression levels of miR-2 and novel-miR-109 were significantly decreased in the ovaries due to JH inhibition. In accordance with previous and current findings, infection with *C*Las or *Cf* led to increased JH titer and reduced abundance of miR-2 and novel-miR-109, ensuring elevated *DcKr-h1* expression, consequently stimulating ovarian development and enhancing fecundity (Fig. 6). These observations highlight the critical roles of miR-2 and miR-109 in the JH-dependent increase in fecundity in psyllids induced by infection with different pathogens. As miRNAs play a crucial role in the interaction between insects and pathogenic microorganisms, for example, the interaction between insects and plants and the interaction between Huanglongbing (HLB) bacteria and citrus, do miRNAs also play an equally important role in a wider range of ecosystems? They are also worth further research. In addition, miRNAs can also be developed as target genes for controlling *D. citri*, thereby blocking the insect vector transmission of HLB. While the infection of *D. citri* with *Cf* pathogens has been observed to increase the number of eggs laid, ongoing investigations are focused on determining whether the hatching rate of these eggs remains unaffected or is reduced.

## MATERIALS AND METHODS

### Host plants and insect colonies

*D. citri* infected with the bacterium, ‘*Candidatus* Liberibacter asiaticus’ (*C*Las+) is named as *C*Las-positive (*C*Las+) *D. citri*. The *C*Las-negative (*C*Las-) and *C*Las +*D. citri* colonies used in this study were obtained from a laboratory culture continuously reared on healthy and *C*Las-infected lemon (*Citrus* ×*limon* (L.) Osbeck) plants. To confirm *C*Las infection status, both the *C*Las +psyllids and lemon plants were subjected to monthly assessment using a quantitative polymerase chain reaction (qPCR) analysis (43). The *C*Las- and *C*Las +*D. citri* populations were reared separately in incubators with similar conditions (26 ± 1°C, 65 ± 5% RH, and a 14L: 10D cycle), while the *C*Las- and *C*Las +lemon plants were kept in different glasshouses.

### Fungal strain and culture

A wild, virulent strain of *C. fumosorosea* (*Cf*) (SCAU-CFDC01, OL872288.1) was collected from an adult cadaver of *D. citri* (44). The fungal isolate was cultivated on Sabouraud dextrose agar with yeast extract at 25 ± 1°C in total darkness for 15 days. Sublethal concentrations (approximating the LC_25_) of conidia were prepared as described in Wu et al. (25). Conidial suspension (20 mL) were evenly sprayed on both sides of the leaves of healthy lemon plants infested with fifth instar nymphs using a manual sprayer (Taizhou Ruipu Gardening Products Co., Ltd). Control leaves were also sprayed with 0.05% Tween-80. Nymphs were observed daily with a stereomicroscope, and emerged adults were transferred to healthy plants for further experiments.

### Assays of reproductive parameters

Adult females (7 days after enclosure (DAE)) from both *C*Las- and *C*Las +colonies, as well as adult females (5 DAE) from control and *C. fumosorosea*-treated plants, were collected for analysis. Female adults from different groups were paired with healthy male adults and placed on young flush of healthy lemon plants for oviposition. The flush with the paired insects was sealed within a tied, white, mesh bag (150 × 200 mm) and placed in an incubator (26 ± 1°C, 65 ± 5% relative humidity [RH], and a 14L: 10D cycle). Preoviposition and oviposition periods were recorded after 24 h, and fecundity (number of eggs laid per female) was assessed. The number of eggs laid by each female was counted daily, and the pairs of psyllids were transferred to a new flush to continue egg-laying. Egg counts were conducted daily until the females died. The experiments were conducted three times, with 15 pairs of psyllids per replication for each infection status.

### Quantitative real time-PCR (qRT-PCR)

To examine the temporal expression profiles of miR-2, miR-109, and *DcKr-h1* in adult female ovaries, adult females at 4 DAE, 8 DAE, and 12 DAE from different colonies were collected and immediately stored at −80°C for total RNA extraction. Total RNA from selected tissues was isolated using TRIzol reagent (Invitrogen, Carlsbad, CA, United States), followed by cDNA synthesis with a PrimeScript II 1st Strand cDNA Synthesis Kit (Takara, Beijing, China). qRT-PCR was performed using TB Green Premix Ex Taq II (Takara, Beijing, China) on an ABI PRISM 7500 Real-Time System (Applied Biosystems, Foster City, CA, USA). For miRNA analysis, miRNAs were extracted using a miRcute miRNA Isolation Kit (TIANGEN, Beijing, China), and cDNA was synthesized using a miRcute Plus miRNA First-Strand cDNA Kit (TIANGEN), with quantification performed using the miRcute Plus miRNA qPCR Kit (SYBR Green) (TIANGEN). *C*Las titers in *C*Las+ psyllids were determined using qRT-PCR as previously reported (45). Internal controls, beta-actin gene (*Dcβ-ACT*, GenBank XM_026823249.1) and U6 snRNA, were used to normalize gene and miRNA concentrations, respectively. Primer sequences for qRT-PCR are listed in Table S1.

### Fluorescence *in situ* hybridization

The ovaries were dissected in 1 × phosphate-buffered saline (PBS) (Beyotime Biotechnology, Shanghai, China) and fixed in Carnoy’s fixative (glacial acetic acid-ethanol-chloroform, 1: 3: 6 (v/v) for 12 h at 25°C. Subsequent steps included rinsing with 6% H_2_O_2_ in 80% ethanol, washing with phosphate buffered solution Triton (PBST, 1 × PBS: TritonX-100, 99.7: 0.3 (v/v)), pre-incubation in hybridization buffer (20 mM Tris-HCl, pH 8.0, 0.9 M NaCl, 30% formamide (v/v), 0.01% sodium dodecyl sulfate (w/v)), and incubation in hybridization buffer containing 10 pmol/mL of each probe for 24 h at 25°C. After staining with 0.1 mg/mL of 40, 60-diamidino-2-phenylindole (DAPI) and mounting, samples were examined using a Leica TCS-SP8 confocal microscope (Leica Microsystems Exton, PA USA) using 405, 488, and 550 nm excitation lasers to detect DAPI, FITC, and Cy3 signals, respectively. The DAPI signals from the cell nuclei were visualized in blue. *DcKr-h1*-FITC, the *DcKr-h1* signals were visualized in green by staining with FITC, and the *C*Las signals were visualized in red by staining with Cy3. Sequential scanning avoided signal overlap. Specificity of detection was carried out without using the probe and *C*Las- psyllids. Image processing was performed using Leica LAS-AF software (v2.6.0). Three FISH tests were conducted per treatment with more than 15 ovaries in total viewed under the microscope. The probe sequences in this study are presented in Table S1.

### Western blotting

Total proteins were extracted from the ovaries of adult females using RIPA protein lysis buffer (50 mM Tris - pH 7.4, 1% Triton X-100, 150 mM NaCl, 1% sodium deoxycholate, 0.1% SDS, and 1 mM PMSF). Equal amounts (50 µg) of protein were separated on 12% SDS-PAGE gels, transferred to PVDF membranes (Millipore), blocked with 5% nonfat powdered milk, and incubated with the primary antibody raised against psyllid *DcKr-h1* (ABclonal Technology Co., Ltd., Wuhan, China, 1: 1,000) for 12 h at 4°C, and the corresponding horseradish peroxidase (HRP)-conjugated secondary antibody (goat anti-rabbit IgG, 1:10,000) for 2 h at 25°C. A mouse monoclonal antibody against β-actin (1:10,000, TransGen Biotech, Beijing, China) was used as a loading control. Bands were imaged using enhanced chemiluminescence with Azure C600 multifunctional molecular imaging system.

### RNAi assay and miRNA treatment

dsRNA of *DcKr-h1* (XM_026820026.1) was transcribed using a Transcript Aid T7 High Yield kit (Thermo Scientific, Wilmington, DE, United States) with a GeneJET RNA Purification kit (Thermo Scientific). miR-2a agomir (agomir-2a), novel-miR-109 agomir (agomir-109), agomir negative control (agomir-NC), miR-2a antagomir (antagomir-2a), novel-miR-109 antagomir (antagomir-109), and antagomir negative control (antagomir-NC) were chemically synthesized and modified by the Shanghai GenePharma Co. Ltd (Shanghai, China). These RNAi and miRNA treatments were performed by feeding dsRNA or miRNA antagomir/agomir through an artificial diet as described previously (19, 25). Briefly, aliquots of twenty female psyllids were placed into a glass cylinders (25 × 75 mm) and sealed with two stretched paramembranes. 200 µL of 20% sucrose (w:v) mixed with dsRNA or miRNA antagomir/agomir was placed between two paramembranes for feeding. The concentrations of ds*DcKr-h1* and miRNA antagomir/agomir were 200 ng/µL and 10 µM, respectively. After feeding with dsRNA or miRNA antagomir/agomir for 48 h, the treated females were assayed as follows. Gene expression levels in the ovaries were assayed using qRT-PCR and western blotting. Reproductive parameters such as pre-oviposition period, oviposition period, and fecundity were recorded. *C*Las titers in the ovaries were determined (45), and ovary morphology was examined using an Ultra-Depth Three-Dimensional Microscope (VHX-500). Green fluorescent protein (GFP) served as a reporter gene, and control groups were fed with ds*GFP* and antagomir-NC/agomir-NC. Each experiment was performed in three replications, and each replication included 15–20 pairs of psyllids.

### Luciferase assay

For software miRanda, the alignment scores of 145 and −15 kcal/mol were set to the threshold. The context score percentile of Targetscan was set as larger than 50 to evaluate the target relationship between miRNA and potential targets. The overlapped miRNAs predicted through both methods were selected. The 3′UTR sequence of *DcKr-h1* (306 bp) containing miRNA-binding sites was cloned into the pmirGLO vector (Promega, Wisconsin, USA) downstream of the luciferase gene to construct the recombinant plasmid, *DcKr-h1*-3′UTR-pmirGLO, using the pEASY-Basic Seamless Cloning and Assembly Kit. For mutation, the seed regions of miR-2 and miR-109-binding sites were mutated. The mutated 3′UTR sequence was amplified and cloned into the pmirGLO vector to generate the *DcKr-h1*-3′UTR mutant-pmirGLO plasmid. The constructed vectors and agomir-2, agomir-109, and agomir-NC were transferred into a human embryonic kidney 293T (HEK293T) cell line in a 24-well plate using a Calcium Phosphate Cell Transfection Kit (Beyotime, Nanjing, China). After 24 h, the luciferase activities were detected using the Dual-Glo Luciferase Assay System (Promega, Madison, WI, USA).

### RNA immunoprecipitation

RIP experiments were performed using a Magna RIP kit (Millipore, Billerica, MA) in accordance with the manufacturer’s protocol. About 50 female psyllids were fed with agomir-2, agomir-109, or agomir-NC for 8 h. Ovaries were then dissected and homogenized in ice-cold RIP lysis buffer, and then those were stored at −80°C overnight. Next, 50 µL magnetic beads were incubated with 5 µg of Ago-1 antibody or normal mouse IgG (Millipore, Billerica, MA) to form bead-antibody complexes. The frozen lysates were centrifuged for 15 min, and the supernatants were divided into three parts. Aliquots (10 µL) of the supernatant of RIP lysate were placed into new tubes and labeled “input”. The other two parts were incubated with the magnetic bead-antibody complex at 4°C overnight. The immunoprecipitated RNAs were released by digestion with protease K. Finally, the precipitated RNAs were eluted and reverse transcribed into cDNA, followed by quantification using qRT-PCR. The abundance of *DcKr-h1* and miRNAs was quantified. The “input” samples and IgG controls were assayed to normalize the relative expression levels of target genes.

### Statistical analysis

Statistical analyses were performed using Student’s *t*-tests or one-way ANOVA followed by Tukey’s honest significant difference (HSD) tests using GraphPad Prism 8.0 software. All statistical values were shown as means ± SEs, and differences were considered significant at *P* < 0.05.

## SUPPLEMENTAL MATERIAL

Supplemental material mbio.01541-24-s0001.pdf Fig. S1-S4; Table S1.

ASM does not own the copyrights to Supplemental Material that may be linked to, or accessed through, an article. The authors have granted ASM a non-exclusive, world-wide license to publish the Supplemental Material files. Please contact the corresponding author directly for reuse.
