# Supplementary material for: The conserved role of miR-2 and novel miR-109 in the increase in fecundity of Diaphorina citri induced by symbiotic bacteria and pathogenic fungi
Source: mBio. 2024 Oct 7;15(11):e01541-24. doi: 10.1128/mbio.01541-24 (PMC11559015; doi:10.1128/mbio.01541-24)
Supplement: Supplemental material — Fig. S1-S4; Table S1. [file mbio.01541-24-s0001.pdf]

The conserved role of miR-2 and novel miR-109 in the increase in fecundity of *Diaphorina citri* induced by symbiotic bacteria and pathogenic fungi

Xiaoge Nian[1,2†], Shujie Wu[1†], Jielan He[1], Paul Holford[3], George Andrew Charles Beattie[3], Desen Wang[1], Yijing Cen[1], Yurong He[1], Songdou Zhang[4*]

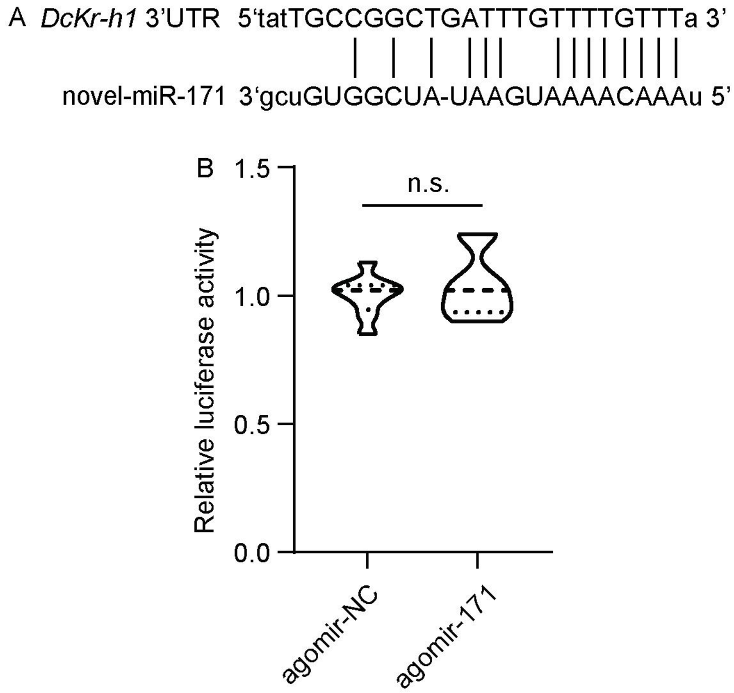

Figure S1. Identification of novel-miR-171 potentially targeting *DcKr-h1.* Related to Figure 1. (A) The putative binding sites of novel-miR-171 in the *DcKr-h1* 3'-UTR as predicted by miRanda and RNAhybrid. (B) Dual-luciferase reporter assays using HEK293T cells co-transfected with agomir-171 and recombinant pmirGLO vectors containing *DcKr-h1*-3'UTR. Data represent three biological replicates with three technical replicates and are shown as means ± SEs. The significant between differences are indicated by asterisks were determined by Student's *t*-tests (*P < 0.05).

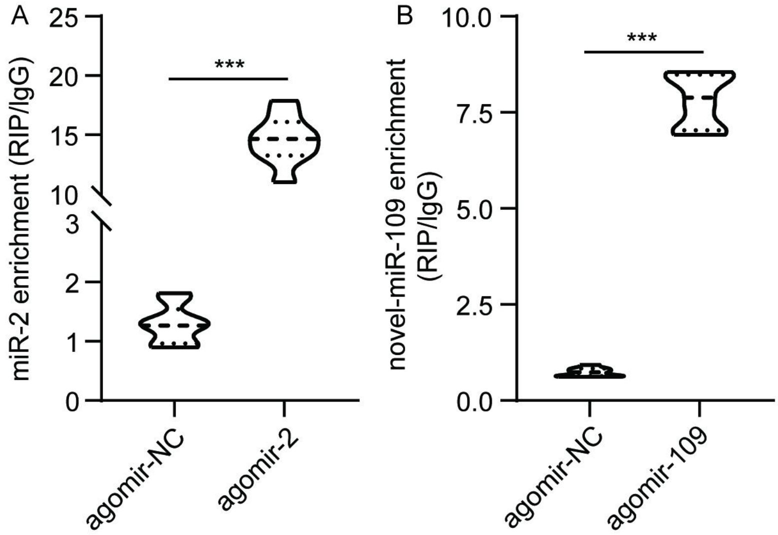

Figure S2. *In vivo* expression of miR-2 and novel-miR-109 in an RNA immunoprecipitation assay. Related to Figure 1. Data represent three biological replicates with three technical replicates and are shown as means ± SEs. The significant between differences are indicated by asterisks were determined by Student's *t*-tests (****P* < 0.001).

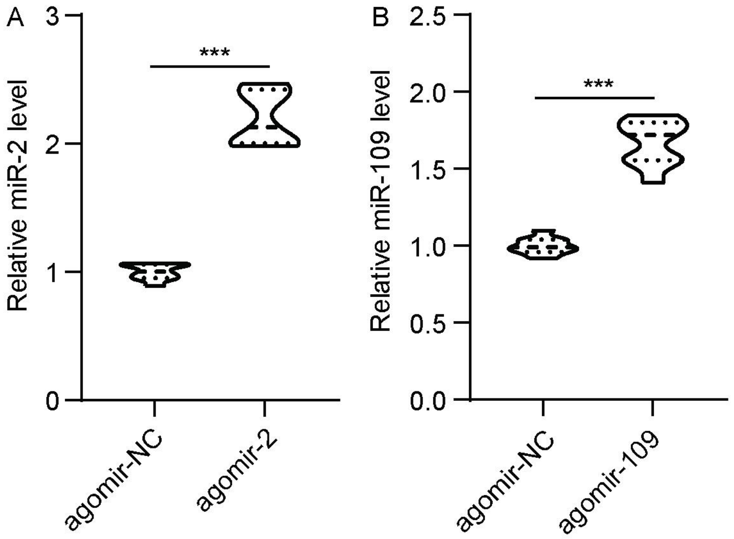

Figure S3. The expression of miR-2 and miR-109 in the psyllids after feeding with agomirs. Related to Figure 1. Data represent three biological replicates with three technical replicates and are shown as means ± SEs. The significant between differences are indicated by asterisks were determined by Student's *t*-tests (****P* < 0.001).

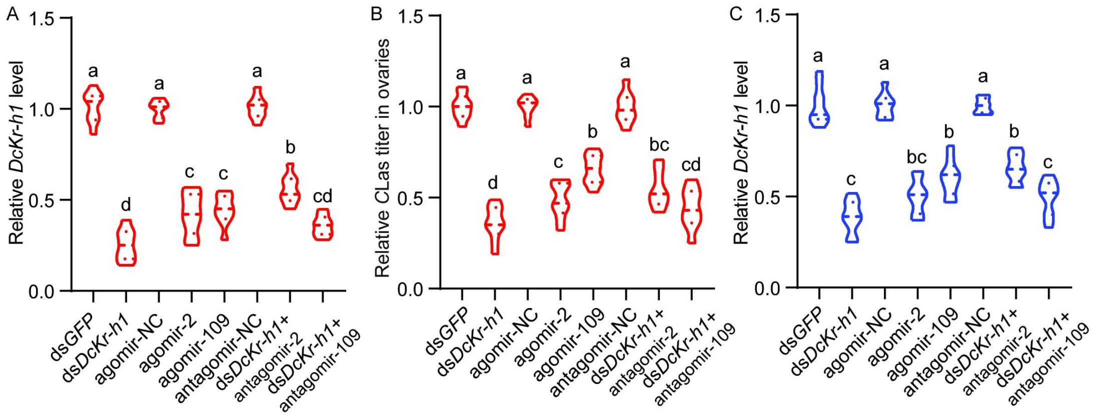

Figure S4. Feeding with antagomirs partially rescues the ovarian defects induced

by feeding with ds*DcKr-h1*. Related to Figure 4. (A-B) Relative expression of *DcKr-h1* and *C*Las titer of *C*Las+ females fed with ds*DcKr-h1*, agomir treatments and antagomir rescues. (C) Relative expression of *DcKr-h1* of *Cf*+ females fed with ds*DcKr-h1*, agomir treatments and antagomir rescues. Data represent three biological replicates with three technical replicates and are shown as means ± SEs. Different letters above the hourglasses indicate significant differences determined by one-way ANOVA followed by Tukey's Honest Significant Difference tests ($P < 0.05$).

Table S1. **Primer list used for qRT-PCR, FISH, and RNAi analysis. Related to Method details.**

Gene name	Accession number	primer name	Sequences of primers (5'-3')	Purpose
DcGFP	ACY56286	*DcGFP*-RNAi-F	taatacgactcactatagggACTCCAGCAGGACCATGTGATC	dsRNA synthesis
		DcGFP-RNAi-R	taatacgactcactatagggACCTGAAGTTCATCTGCACCAC	
Dcβ-ACT	DQ675553.1	*Dcβ-ACT*-qF	TGTTCCAACCTTCCTTCCTG	qRT-PCR
		Dcβ-ACT-qR	GTGTTGGCGTACAGGTCCTT	
		DcMet-RNAi-F	taatacgactcactatagggCTTCATCTGTCGTATCAAGGT	dsRNA synthesis
		DcMet-RNAi-R	taatacgactcactatagggATGAACTCTGCGTTGTTACT	
DcKr-h1	XM_026820026.1	*DcKr-h1*-qF	CTCCAGTGCTGAGTCCACAA	qRT-PCR
		DcKr-h1-qR	ATCTCCCGGAGGTTTCTGTT	
		DcKr-h1-RNAi-F	taatacgactcactatagggGACGGAACCTCCAAGTTCAAAA	dsRNA synthesis
		DcKr-h1-RNAi-R	taatacgactcactatagggGAGTGTGGTGAAGTTTCGCCTT	
		DcKr-h1-3'UTR-F	GCTGTGAGGTATAGTTCTG	3'UTR amplification
		3'RACE-Outer Primer	TACCGTCGTTCCACTAGTGATTT	
		DcKr-h1-3'UTR-Full-F	CTAGTTGTTTAAACGAGCTCTGCAAAGCCTCCTTTAAGGC	Full sequence of 3'UTR
		DcKr-h1-3'UTR-Full-R	TGCATGCCTGCAGGTCGACTCTAGATACCGTCGTTCCACTAGTGA	
		DcKr-h1-3'UTR-mutant (miR-2)-F	CTAGTTGTTTAAACGAGCTCTGCAAAGCCTCCTTTAAGGC	Mutant sequence of 3'UTR
		DcKr-h1-3'UTR-mutant (miR-2)-R	TGCATGCCTGCAGGTCGACTCTAGAATACAAACTAGACCAGAAG	
		DcKr-h1-3'UTR-mutant (miR-109)-F	CTAGTTGTTTAAACGAGCTCTTGTAAACATACAAACATCCC	Mutant sequence of 3'UTR
		DcKr-h1-3'UTR-mutant (miR-109)-R	TGCATGCCTGCAGGTCGACTCTAGATACCGTCGTTCCACTAGTGA	

		DcKr-h1-probe	FITC-UGCCAUGCUAUCAGGUAUA AGAUAAACAUUAGGCUCAG	Labled with FITC for FISH
CLas 16s rRNA	L22532.1	*C*Las 16s-probe	Cy3-CATTATCTTCTCCGGCG	Labled with Cy3 for FISH
		*C*Las 16s-qF	TCGAGCGCGTATGCAATACG	qRT-PCR
		*C*Las 16s-qR	GCGTTATCCCGTAGAAAAAGGTAG	
miR-2	-	miR-2-qF	TATCACAGCCAGCTTTGATGAGC	qRT-PCR
novel-miR-109	-	novel-miR-109-qF	TAAACAAAACCCATTTCGGCACTG	qRT-PCR
U6	-	U6-qF	AGGATGACACGCAAAATCGT	qRT-PCR

Note: The boxed sequences indicated the homologous arm sequence used for vector seamless clone.
